# Editorial: Nutriomics in livestock research

**DOI:** 10.3389/fvets.2022.1071290

**Published:** 2022-11-15

**Authors:** Rajib Deb, Rita Payan-Carreira

**Affiliations:** ^1^Animal Health Division, Indian Council of Agricultural Research (ICAR)-National Research Center on Pig, Guwahati, India; ^2^Comprehensive Health Research Centre and Department of Veterinary Medicine, University of Evora, Évora, Portugal

**Keywords:** nutriomics, livestock, feeding, productivity, vaccine

The development of high-throughput technology offers robust platforms to measure the components of the gut microbiota as well as proteins, metabolites, and coding and non-coding RNAs. Transcriptomics, proteomics, metabolomics, and metagenomics are just a few of the technologies that can be used to gather information about the genome and other biological molecules, and they are all effectively applied in the field of nutrigenomics, also known as nutritionomics, to shed light on the interactions between nutrition and the livestock genome. Integrating genomics and nutrition facilitates understanding the differences or similarities in how different candidate genes express themselves in response to food. Since nutriomics is still in its infancy, it presents an exciting potential in animal nutrition to ensure the quality of nutrition and its impact on general improvement of animal production. This Research Topic aims to assemble papers examining the effects of various diets (and/or nutrients) while feeding livestock, using nutriomics to increase productivity, enhance reproductive parameters, and prevent or modify livestock disease prevention. The nine publications that integrate this unique e-collection discuss various nutriomics topics in livestock.

From a nutrigenomic perspective, dairy cows' metabolic difficulties between conception and lactation is of great interest. A state of energetic insufficiency is driven by a decline in feed intake and a sudden rise in energy demands caused by the quickly shifting of the cow metabolic landscape. The mobilization of non-esterified fatty acids (NEFA), which are used as energy, balances this situation. The Peroxisome Proliferator-Stimulated Receptor (PPAR), a transcriptional regulator with established nutrigenomic characteristics, is interestingly activated by peripartum NEFA. Precision-cut liver slices (PCLS) from liver biopsies were studied by Busato et al. as a model for PPAR activation in periparturient dairy cows. A subset of 91 genes among the differentially expressed genes in the bovine liver was been identified as potential novel PPAR targets.

Understanding the biological mechanisms governing feed efficiency using a readily available and non-invasive sample, like as blood, is crucial for the future of livestock production systems regarding profitability and animal welfare concerns. Critical pathways related to beef cattle's residual feed intake (RFI) were identified using the gene set enrichment technique to analyze whole blood transcriptome data. Such finding revealed that depending on whether beef steers were divergently selected for low or high RFI, the expression of genes involved in protein metabolism and stress response varied (Taiwo et al.).

In the USA, intramuscular fat deposition or marbling is a key factor in determining the quality and worth of the carcass. The transient rise in intramuscular fat deposition suggests the involvement of an epigenetic adaptation. Bta-miR-122 was identified by small RNA sequencing as a possible miRNA of interest that may be related to intramuscular fat accumulation with rising time-on-concentrates (TOC). Using real-time ultrasound measurements of intramuscular fat, the authors suggest that preadipocyte differentiation may first be stimulated, triggering then a global up-regulation of lipogenic genes involved in *de novo* fatty acid synthesis, which provides fatty acids for subsequent hypertrophy. This hypothesis is supported by differential gene expression and increased intramuscular fat content (Duckett and Greene).

Wang X. et al. coupled multi-omics analyses with microbiome and metabolomics research to assess the impact of dietary protein levels on Tibetan sheep ruminal microbial communities and metabolites. According to this study, the optimal dietary protein intake for Tibetan sheep during the winter season is between 12.1 and 14.1% protein. This research improved our knowledge of ruminal microbial and metabolic processes and may influence the amount of protein needed in Tibetan sheep diets and of the nutritional control.

Allium mongolicum (AMO) Regel essential oil's effects on sheep growth performance, nutritional digestibility, rumen fermentation, and bacterial populations were studied by Yaxing et al. This study found higher cellulase, beta-amylase, and proteinase activity levels existed in the AMO group compared to the control group (*P* < 0.05). Dry matter (DM) and crude protein (CP) appeared to be more digestible in the AMO group than in the control group (*P* < 0.05). In conclusion, using AMO supplements may enhance growth efficiency. AMO supplementation also improved the rumen fermentation, bacterial populations, and nutrient digestibility in sheep.

By comparing the effects of food supplementation with and without *Bacillus subtilis* (*B. subtilis*), Tang X. et al. looked at how broiler chicken carcass characteristics, meat quality, amino acids, and fatty acids were affected. The findings indicated that adding *B. subtilis* to the feed could enhance broilers' meat quality and carcass characteristics. This would be advantageous for customers given the improved fatty acid profile and amino acid composition.

A significant commercial crop, soybean is the primary plant protein source for food, feed, and other industries. One of the eight allergenic foods, soybeans have the potential to negatively impact animal growth and health as well as the respiratory and digestive systems. The primary antigenic element of soybean protein is 11S glycinin. Wang L. et al., demonstrated that 11S Glycinin up-Regulated NLRP-3-Induced Pyroptosis by Triggering Reactive Oxygen Species (ROS) in Porcine Intestinal Epithelial Cells.

Lactobacillus plantarum (*L. plantarum*) CGMCC 1258 and Lactobacillus reuteri (*L. reuteri*) LR1 are two significant probiotic strains. According to research done by Tang Q. et al., dietary *L. reuteri* LR1 resulted in better growth performance, a lower incidence of diarrhea, better intestinal morphology, and a higher degree of immune activation in weaned pigs, while dietary *L. plantarum* CGMCC 1258 improved intestinal morphology, intestinal permeability, intestinal immunity, and antioxidant function in weaned pigs.

Shao et al. investigated how dietary selenium homolanthionine (SeHLan) affected the immune system and antioxidant status in puppies receiving canine parvovirus (CPV) vaccinations. Their research discovered that dietary SeHLan supplementation promoted immunological function, elevated CPV antibody titers, and improved antioxidant activity in puppies after weaning. A dose of SeHLan of 12 mg/kg DM in puppies may benefit their nutrition.

The articles on this Research Topic offer superb illustrations of nutriomics investigations and their significance for the wellbeing and output of livestock.

## Author contributions

All authors listed have made a substantial, direct, and intellectual contribution to the work and approved it for publication.

## Conflict of interest

The authors declare that the research was conducted in the absence of any commercial or financial relationships that could be construed as a potential conflict of interest.

## Publisher's note

All claims expressed in this article are solely those of the authors and do not necessarily represent those of their affiliated organizations, or those of the publisher, the editors and the reviewers. Any product that may be evaluated in this article, or claim that may be made by its manufacturer, is not guaranteed or endorsed by the publisher.

